# Bacterial diversity in primary infected root canals of a Chinese cohort: analysis of 16 S rDNA sequencing

**DOI:** 10.1186/s12903-023-03618-3

**Published:** 2023-11-27

**Authors:** Ziqiu Hu, Yonggang Xiang, Yanhong Wei, Xinsheng Gu, Weidong Leng, Lingyun Xia

**Affiliations:** 1 Department of Stomatology, Taihe Hospital, Hubei University of Medicine, Shiyan, 442000 China; 2 Institute of Oral Diseases, School of Dentistry, Hubei University of Medicine, Shiyan, 442000 China; 3 Department of Ophthalmology, Taihe Hospital, Hubei University of Medicine, Shiyan, 442000 China; 4 Department of Pharmacology, School of Basic Medical Sciences, Hubei University of Medicine, Shiyan, China

**Keywords:** Bacterial diversity, 16 S rDNA, Primary endodontic infection, Chronic periapical periodontitis, Root canal

## Abstract

**Purpose:**

To characterize the bacterial community in the primarily infected root canals.

**Methods:**

A total of 13 samples were collected from the primarily infected root canals. 16 S rDNA sequencing was performed to define bacterial community. Taxonomic annotation, bacterial hierarchical structures, community richness and diversity, and inter-subject variability of the bacterial community in the root canal samples were analyzed. Gender, age, and duration of the toothache-specific bacterial community associated with the patient groups were analyzed.

**Results:**

A total of 359 Species were annotated and identified in the whole study cohort. The Alpha diversity analysis showed that the species diversity and detection rate of the 13 samples were high, which reflected the authenticity of sequencing results. The Beta diversity analysis was used to compare the degree of difference between different root canal samples. The 13 samples were divided into two groups according to the results, group A was samples I1-I12, and group B was samples I13. The bacterial species of group A samples were analyzed with the clinical characteristics of patients, and it was found that gender, and duration specific differences in bacterial species, and there was no significant difference in species types among different ages of patients.

**Conclusion:**

There were a wide diversity and inter-subject variability in the bacterial community in the primary infected root canals. While *Porphyromonas gingivalis* was the most abundant species, *Fusobacterium nucleatum* was the most variable species in the bacterial community of the root canal. The bacterial community at different taxonomic levels varied from sample to sample, despite consistent disease diagnoses. There was gender, duration-specific differences in the bacterial species in the primary infected root canals.

**Supplementary Information:**

The online version contains supplementary material available at 10.1186/s12903-023-03618-3.

## Introduction

Root canal treatment is often applied to patients with infection in the pulp and/or periapical tissue to eliminate infection, preserve natural teeth, and maintain systemic health. The root canal infection may result from the deep decay, faulty crown, crack, or chip in the tooth, where the microbes are implanted, localized, proliferated, and disseminated. In addition, agents such as lipopolysaccharide (LPS) produced by the microbes and the proinflammatory factors produced from interaction between the microbes and host contribute polymicrobial etiology to the endodontic infections and lead to clinical features [1]. Establishment of association of the microbial factors with clinical symptomatology is crucial to apply appropriate therapeutic procedures for a more predictable outcome of endodontic treatment.

Primary endodontic infection is characterized by wide bacterial diversity and a high inter-subject variability [2, 3]. Using clonal analysis, Nobrega and Montagner et al. identified 76 phylotypes, of which 47 (61.84%) are different species and 29 (38.15%) are taxa reported as yet-uncultivable or as yet-uncharacterized species. *Prevotella spp.*, *Fusobacterium nucleatum*, *Filifactor alocis*, and *Peptostreptococcus stomatis* are the most frequently detected species, followed by *Dialister invisus*, *Phocaeicola abscessus*, *the uncharacterized Lachnospiraceae oral clone*, *Porphyromonas spp.*, and *Parvimonas micra*. Eight phyla were detected and the most frequently identified taxa is the phylum Firmicutes (43.5%), followed by Bacteroidetes (22.5%) and Proteobacteria (13.2%) [3]. There are about 110 species of bacteria in the infected root canals, obligate anaerobe and Gram-negative bacteria are prevalent, 258 groups have been identified by microbial culture techniques, and 317 groups have been identified by molecular biology methods [4, 5]. In addition, different pathological types of periapical lesions have different bacterial species [6–8]. The different clinical and imaging features of periapical periodontitis patients may be related to bacterial diversity [9]. However, few studies have analyzed the degree of variation in microbiota between samples and related microbiota characteristics to patient’s age, gender, and pain duration.

There are a large number of unculturable microorganisms in the infected root canals, and a low abundance of bacteria may play an important role in the pathogenic bacteria [10–12], compared with species diversity in infected root canals studied mainly by culture [13]. The 16 S rDNA high-throughput sequencing has become a common method for community species identification and classification, which can provide more accurate and reliable identification of bacteria that are difficult to identify or cannot be identified by phenotypic experiments [14, 15]. The DNA corresponding to 16 S rRNA has a moderate molecular weight and is highly conserved in structure, which improves the accuracy of species identification while maintaining the privacy of samples. It can not only reflect the similarity between species but also reflect the differences between species [16]. Therefore, characterized the bacterial community in the primary infected root canals is contribute to explore the structure and changes of bacterial communities, and provides a theoretical basis for the screening and confirmation of pathogenic strains. Clinically, it could more helpful to understand and control root canal infection and inflammation, and improve the success rate of root canal treatment.

In this study, we employed 16 S rDNA sequencing to characterize the bacterial community of the primary infected root canals, taxonomic annotation, bacterial hierarchical structures, community richness and diversity, and inter-subject variability of the bacterial community in the root canal samples were analyzed. Gender, age, and duration of the toothache-specific bacterial community associated with the patient groups were analyzed. Our study may provide data to understand pathogenesis and development of conditions such as primary apical periodontitis.

## Materials and methods

### Patients

Inclusion criteria: Patients referred treatment with facial swelling or painful teeth in the Department of Stomatology, Taihe Hospital, Hubei University of Medicine, and diagnosed with chronic periapical periodontitis after physical examination (pain on palpation, tenderness to percussion and persistent sinus tract, etc.) and imagological examination (the presence of persistent periapical radiolucent lesions, diameter less than 5 mm). The diagnosed teeth had no history of root canal treatment.

Exclusion criteria: Patients received antibiotics within three months before this treatment, or have chronic periodontitis (defined as clinical attachment loss ≥ 6 mm or probing depth ≥ 5 mm at two or more dental positions in the mouth). The patients with systemic diseases such as diabetes, cardiovascular disease, atherosclerosis, and stroke, HIV/AIDS, rheumatoid arthritis, or any other systemic disease that impairs the immune system.

### Sample collection and DNA extraction

Patient were instructed to gargle with 0.2% chlorhexidine for 3 min. After standardized sterilization of the affected tooth and its surroundings using 1% iodine tincture, rubber dam was used to isolate the affected teeth. The enamel, dentin, or residual filling material of the affected tooth were removed using sterile high-speed handpiece and carbide burs cooled with sterile water. The top of the pulp chamber is removed with a sterile round bur in anhydrous condition. The root canals were examined using a sterile probe and cleared using a size 15-K file. The walls of root canals were repeatedly filed after determination of the working length using the electronic apex locator. The fluids in the processed root canals were absorbed for 30 s using a sterile paper point. 200 μL sterile saline was dropped into the root canal and collected using another sterile paper point. The process was repeated another time. All three sample collection paper tips and the size 15-K file tip were collected into a 1.5 mL centrifuge tube containing 1 mL TE buffer and labeled. The tubes were gently shaken and stored in -80℃. Total DNAs in the samples were extracted using PowerSoil® DNA Isolation kit (Mo Bio Laboratories) and quantified using a microplate reader (SynergyHTX, Gene Company Limited) [4, 15, 17].

### 16 S rDNA sequencing and data preprocessing

DNA library for sequencing was constructed through SolexaPCR using the barcoded universal primers 27 F (16 S-F): AGRGTTTGATYNTGGCTCAG 1492R (16 S-R): TASGGHTACCTTGTTASGACTT (amplicon 1466 bp) and total DNA samples. After PCR amplification, the products were purified, quantified, and homogenized into the SMRTBell sequencing library. After quality inspection, the library was subjected to PacBioSequel sequencing. CCS file obtained from raw PacBioSequel sequencing data using smrtlink software were processed by Lima v1.7.0 software to identify specific Raw-CCS sequences of all samples. The Clean-CCS sequences were obtained after removing the primer sequences and length filtering using cutadapt 1.9.1 software. The Effective-CCS sequence data were obtained for subsequent analysis after removal of the chimeric sequences using UCHIME v4.2 software [18]. The effective rate was defined as the ratio of the Clean-CCS sequences relative to the Raw-CCS sequences.

### Taxonomic annotation, bacterial hierarchical structures, community richness and diversity

The effective CCS sequences were clustered, OTU was divided, and species classification was obtained according to the sequence composition of OTU. Based on the results of OTU analysis, the samples were analyzed at different taxonomic levels, and the community structure maps and phylogenetic trees of each sample at different taxonomic levels were obtained. Alpha diversity analysis was used to study the species diversity within a single sample, which was measured by Chao1, Ace, Simpson, Shannon, and Coverage index [19]. Rarefaction curves, Shannon index curve, and rank abundance curve was plotted to examine the relationship between bacterial abundance and diversity, evenness in infected root canal samples, or sampling intensity [20, 21].

The cumulative curve reflected the relationship between the number of samples and the number of species. In the curve, a single red box reflects the total number of species contained in the samples, and the total red box forms a cumulative curve, reflecting the rate of new species under continuous sampling. Within a certain range, with the increase of sample size, if the curve shows a sharp rise, it means that many new species have been found in the colony. When the curve tends to be flat, it means that the species in this environment will not increase significantly with the increase of sample size. A single green box reflects the number of species in common in the samples; The total green box shaped composition of the common species curve reflects the rate of occurrence of common species in the sample under continuous sampling. Within a certain range, with the increase of the sample size, if the curve shows a decline, it means that the newly discovered common species in the sample are gradually decreasing. When the curve tends to be flat, it means that the common species in the environment tend to be saturated. The cumulative curve of species and functions can be used to judge whether the sample size is sufficient. The sharp rise of the curve indicates that the sample size is insufficient and needs to be increased; On the contrary, it indicates that the sampling is sufficient for data analysis.

### Inter-subject variability of the bacterial community in the root canal samples

The differences in species diversity (community composition and structure) among different samples were compared through beta diversity analysis using QIIME versions 2 based on the Binary-Jaccard algorithm and the Unweighted Unifrac algorithm [22, 23]. To reveal the similarity and differences in the bacterial communities among the 13 primary endodontic samples, phylogenetic analysis using UPGMA (Unweighted Pair-group Method with Arithmetic Mean) and abundance histogram were performed [24, 25].

### Gender, age, duration of toothache-specific bacterial community associated with the root canal samples

The patients were divided into the male and female groups, the younger ( = < 36Y4M) and older ( = > 38Y6M) group, the group with short-duration toothache ( = < 1 week) and the group with long-duration toothache ( = > 1 month). The differences in the abundance of each species between two groups were statistically compared using T-test. P < 0.05 were considered significant.

## Results

### Samples and rDNA sequencing

**Fig. 1:**
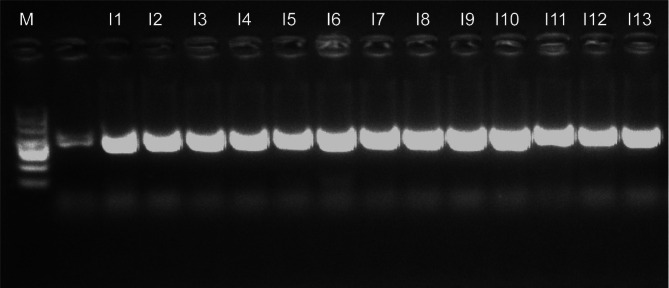
DNA fragments amplified by PCR using DNA samples from 13 root canal patients and universal primer pair 27 F and 1492R

A total of 13 patients subjected to root canal were included in this study (Table 1). DNA samples were extracted from samples rinsed through the root canals of 13 patients. The PCR products were obtained using universal primer pair 27 F and 1492R (Fig. 1) and subjected to 16 S RNA sequencing analysis. A total of 78,808 raw-CCS DNA fragments in the length of 1446–1466 bp for all samples were obtained from sequencing, with an average of 6062, range from 3836 to 7489, sequences per sample. After cleaning and filtering, a total of 73,007 effective-CCS sequences for all samples (mean 5615, range from 3555 to 7035) were obtained. The effective rate for each sample was > 91% (Table 2). Therefore, the effective-CCS sequences were qualified for subsequent data analysis.

**Table 1:** Demographic and clinical characteristics of the patients

Samples	Gender	Age	Diagnosis	Symptom	History
I1	Female	55Y10M	34 Chronic periapical periodontitis	Toothache for more than 1 week	NA
I2	Male	38Y6M	43 Chronic periapical periodontitis	Toothache for several days	NA
I3	Female	72Y4M	24 Chronic periapical periodontitis	Repeated swelling and toothache for more than 1 month	NA
I4	Male	58Y6M	41 Chronic periapical periodontitis	Gingival sinus tract for 4 days	NA
I5	Female	15Y4M	46 Chronic periapical periodontitis	Repeated toothache for more than 1 month	NA
I6	Female	55Y10M	27 Chronic periapical periodontitis	Toothache for 1 week	NA
I7	Female	28Y2M	36 Chronic periapical periodontitis	Toothache for more than 1 week	NA
I8	Male	46Y0M	34 Chronic periapical periodontitis	Toothache for 1 month	HBV
I9	Female	31Y0M	16 Chronic periapical periodontitis (cracked teeth)	Toothache 4 days	NA
I10	Male	16Y10M	15 Chronic periapical periodontitis	Repeated toothache for more than half a year	NA
I11	Male	18Y4M	21 Chronic periapical periodontitis	Tooth discoloration for more than 1 year	NA
I12	Male	36Y4M	12 Chronic periapical periodontitis	Pain on palpation and repeated swelling for more than 1 year	NA
I13	Male	30Y8M	21 Chronic periapical periodontitis	Discomfort with chewing for more than 1 week	NA

**Table 2:** Total number of samples and total sequences processed

Sample ID	Raw CCS	Clean CCS	Effective CCS	AvgLen(bp)	Effective(%)
I1	3836	3588	3555	1461	92.67
I2	6029	5670	5648	1458	93.68
I3	6026	5592	5565	1455	92.35
I4	7473	6985	6892	1464	92.25
I5	7018	6493	6430	1456	91.62
I6	6081	5713	5688	1458	93.54
I7	6047	5605	5542	1461	91.65
I8	7489	6940	6901	1454	92.15
I9	5513	5156	5118	1458	92.84
I10	7489	7055	7035	1466	93.94
I11	4655	4330	4277	1465	91.88
I12	6197	5736	5698	1458	91.95
I13	4955	4672	4658	1446	94.01

### Bacterial hierarchical structures and community richness and diversity in the root canal samples

**Fig. 2:**
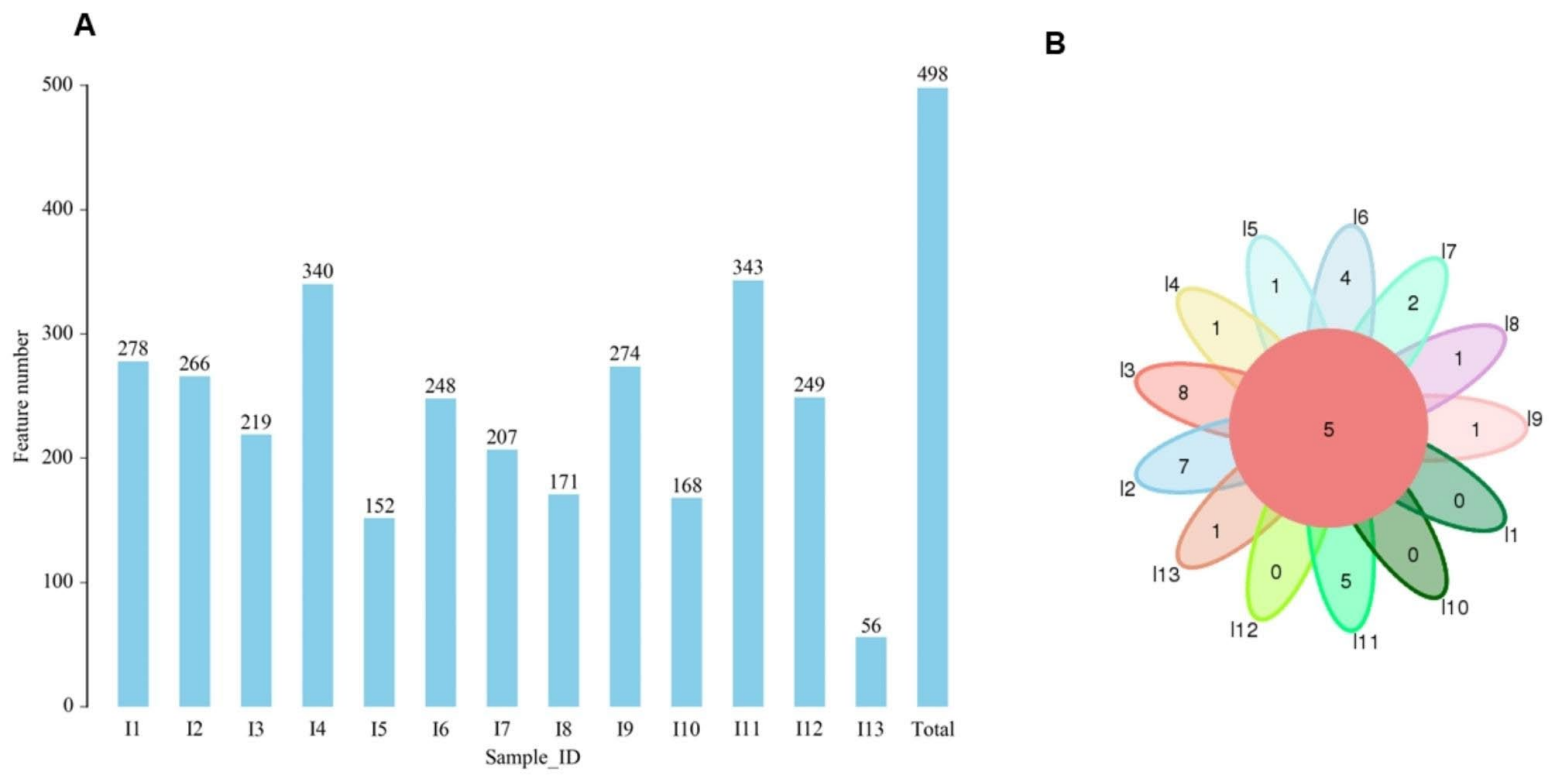
The numbers of OTUs in the root canal sample. (**A**) The numbers of OTUs in the root canal samples. (**B**) The common OTUs in the samples

To identify OTUs, we clustered the effective CCS at the similarity level of 97.0%, and a total of 498 types of OTUs were identified from all 13 samples, with an average of 228 per sample, range from 56 to 343 (Fig. 2A). A total of five common OTUs were identified in all samples (Fig. 2B).

**Fig. 3:**
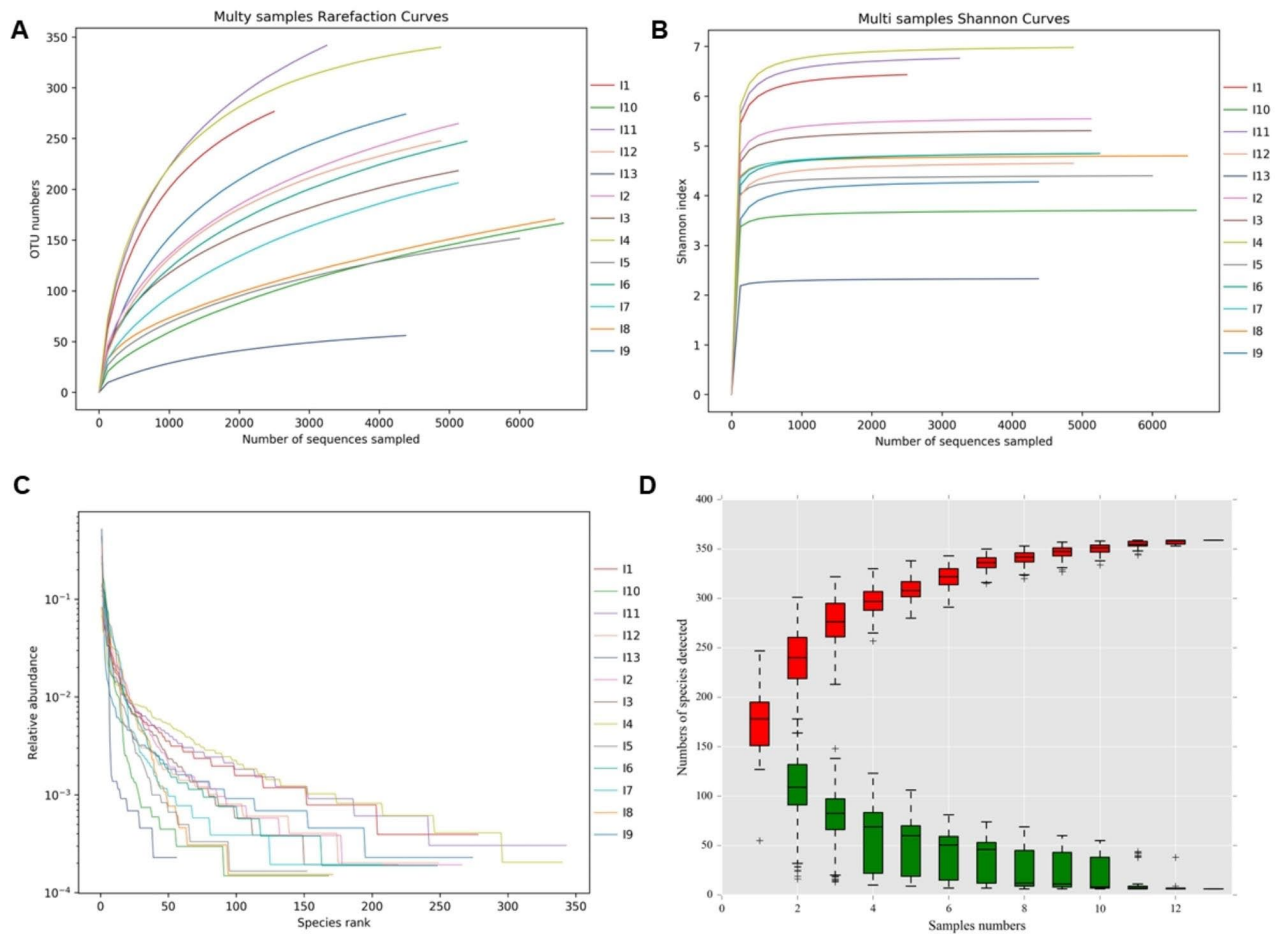
Alpha diversity analysis. (**A**) Rarefaction curves of 16 S rDNA gene sequences for each sample calculated for OTUs at 97% similarity. Vertical axis shows operational taxonomic units, and horizontal axis shows the number of samples sequenced. OTU is operational taxonomic units. (**B**) Shannon index curve. (**C**) Hierarchical abundance curve. (**D**) The cumulative curve of relative abundance of species reflects the relationship between the number of samples and the number of species noted

We analyzed alpha diversity of each sample. The rarefaction curve, Shannon index curve, and rank abundance curve are shown in Fig. 3, which indicated the relationship between bacterial abundance and diversity, evenness in infected root canal samples, or sampling intensity. The results showed that the discovery of OTUs in all samples were saturated and exhausted by the sequencing analysis in this study as indicated by the Rarefaction curves and Shannon index curve (Fig. 3A and B). The numbers of types of bacteria in the samples were between 69.91 and 434.98 as indicated by the Chao1 values, and the values were between 68.78 and 438.58 as revealed by the ACE values (Table 3). The diversity and evenness of the bacteria community were high as indicated by the Shannon index and Simpson indexes (Table 3; Fig. 3C). The coverage rates were all higher than 95% (Table 3), indicating a high probability of species being detected in the samples and reflecting the authenticity of the sequencing results. The cumulative curve reflecting the relationship between the number of samples and the number of species (Fig. 3D) suggested that both the number of samples and the number of species in this study were saturated and therefore sufficient for data analysis.

**Table 3:** Community richness and diversity

Sample ID	Feature	ACE	Chao1	Simpson	Shannon	Coverage
I1	278.00	335.44	330.36	0.97	6.43	0.97
I2	266.00	367.11	349.32	0.95	5.55	0.98
I3	219.00	300.62	280.92	0.95	5.31	0.99
I4	340.00	362.51	359.41	0.98	6.98	0.99
I5	152.00	228.57	207.10	0.92	4.40	0.99
I6	248.00	340.70	327.46	0.90	4.85	0.98
I7	207.00	315.63	282.62	0.94	4.84	0.98
I8	171.00	438.58	267.87	0.94	4.80	0.99
I9	274.00	338.60	347.49	0.78	4.28	0.98
I10	168.00	362.44	251.42	0.87	3.71	0.99
I11	343.00	437.44	434.98	0.98	6.76	0.97
I12	249.00	316.09	324.00	0.85	4.65	0.98
I13	56.00	68.78	69.91	0.68	2.33	1.00

Community richness indices: Feature, ACE, Chao1. Simpson: Simpson diversity index; Shannon: Shannon diversity index

**Fig. 4:**
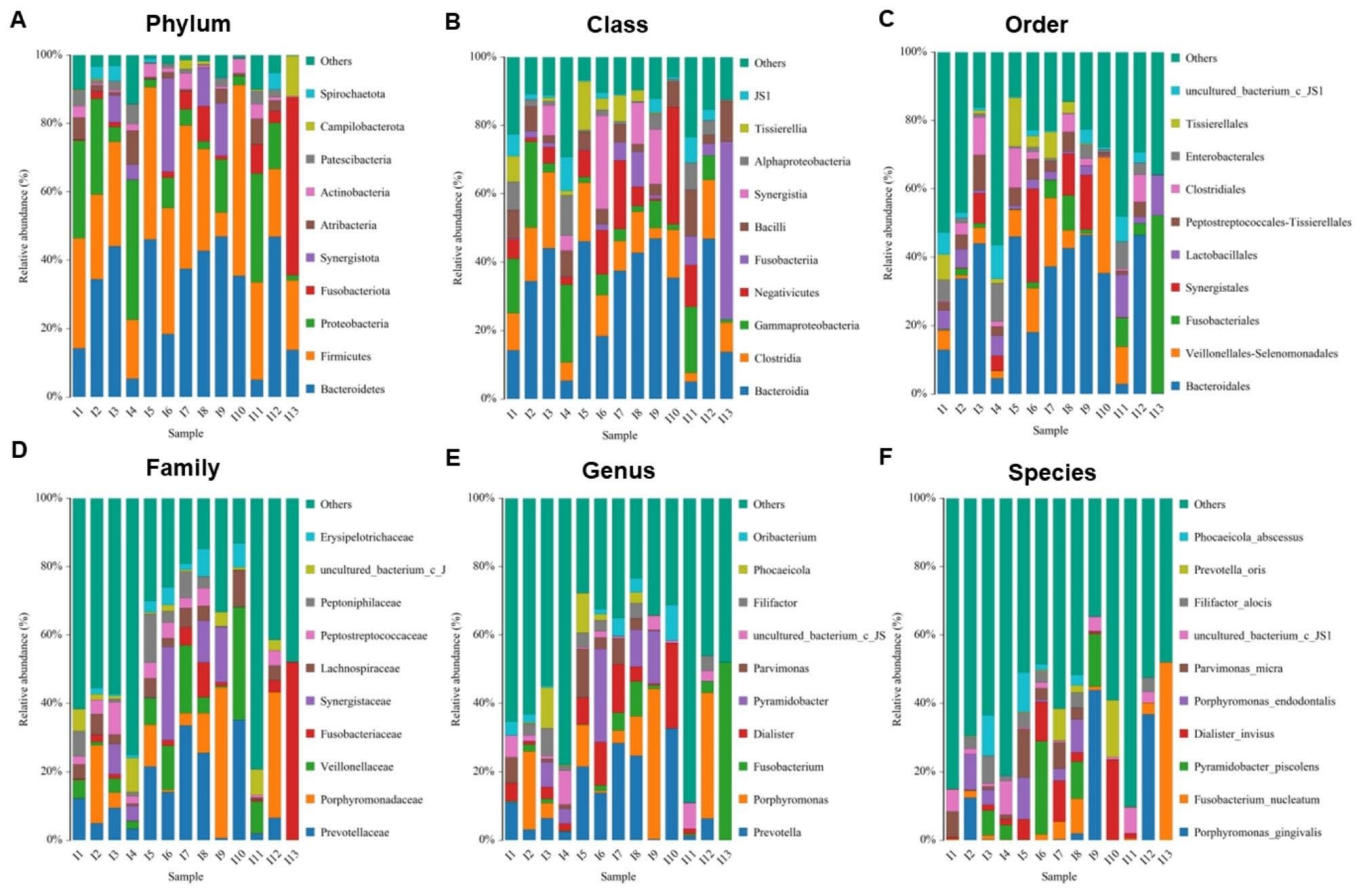
Taxonomic annotation and the hierarchical structures of the bacterial community in the root canal samples. (**A**) Phylum, (**B**) Class, (**C**) Order, (**D**) Family, (**E**) Genus, and (**F**) Species. One color represents an OTU, and the length of the color block represents the relative abundance proportion of species. Only the top ten species in the abundance level are displayed, and other OTUs are combined into Others and displayed in the figure

With taxonomic annotation, the hierarchical bacterial community contained a total of 40 Phyla, 63 Classes, 124 Orders, 180 Families, 265 Genera, 359 Species in the whole study samples (Table 4). The ranges of Phylum 10–40, Class 13–63, Order 25–124, Family 36–180, Genus 47–265, Species 55–359 were detected in the different root canal samples. The most abundant phylum was Bacteroidetes (32.05%), followed by Firmicutes (31.23%), Proteobacteria (12.49%), Fusobacteriota (6.19%), Synergistota (5.62%), Atribacteria (2.42%), and Actinobacteria (2.08%) (Fig. 4A). The most abundant Class was Bacteroidia (32.05%), followed by Clostridia (12.09%), Gammaproteobacteria, Negativicutes, Fusobacteriia, Bacilli, Synergistia, and Tissierellia (Fig. 4B). The most abundant Order was Bacteroidales, followed by Veillonellales-Selenomonadales, Fusobacteriales, Synergistales, Lactobacillales, Peptostreptococcales-Tissierellales, and Clostridiales (Fig. 4C). The most abundant Family was Prevotellaceae, followed by Porphyromonadaceae, Veillonellaceae, Fusobacteriaceae, Synergistaceae, Lachnospiraceae, and Peptostreptococcaceae (Fig. 4D). The most abundant Genus was Prevotella, followed by *Porphyromonas*, *Fusobacterium*, *Dialister*, *Pyramidobacter*, *Parvimonas* (Fig. 4E). The most abundant Species was *Porphyromonas gingivalis*, followed by *Fusobacterium nucleatum*, *Pyramidobacter piscolens*, *Dialister invisus*, *Porphyromonas endodontalis*, and *Parvimonas micra* (Fig. 4F). There was a wide diversity in the bacterial community in the root canals. The bacterial community structure maps at different taxonomic levels suggested that species composition varies from sample to sample, despite consistent disease diagnoses.

**Table 4:** Taxonomic annotation of OTUs and hierarchical structures in all 13 endodontic root canal samples

Sample	Kindom	Phylum	Class	Order	Family	Genus	Species
I1	1	34	52	92	124	160	195
I2	1	32	49	89	123	166	210
I3	1	31	44	74	106	146	178
I4	1	36	59	108	141	186	219
I5	1	22	38	59	79	107	127
I6	1	28	50	84	112	151	190
I7	1	27	43	73	94	126	163
I8	1	26	40	69	92	119	151
I9	1	32	49	91	126	159	188
I10	1	22	38	65	88	116	133
I11	1	34	55	107	143	193	247
I12	1	30	47	87	118	148	170
I13	1	10	13	25	36	47	55
Total	1	40	63	124	180	265	359

### Inter-subject variability of the bacterial communities in the primary endodontic root canal samples

**Fig. 5:**
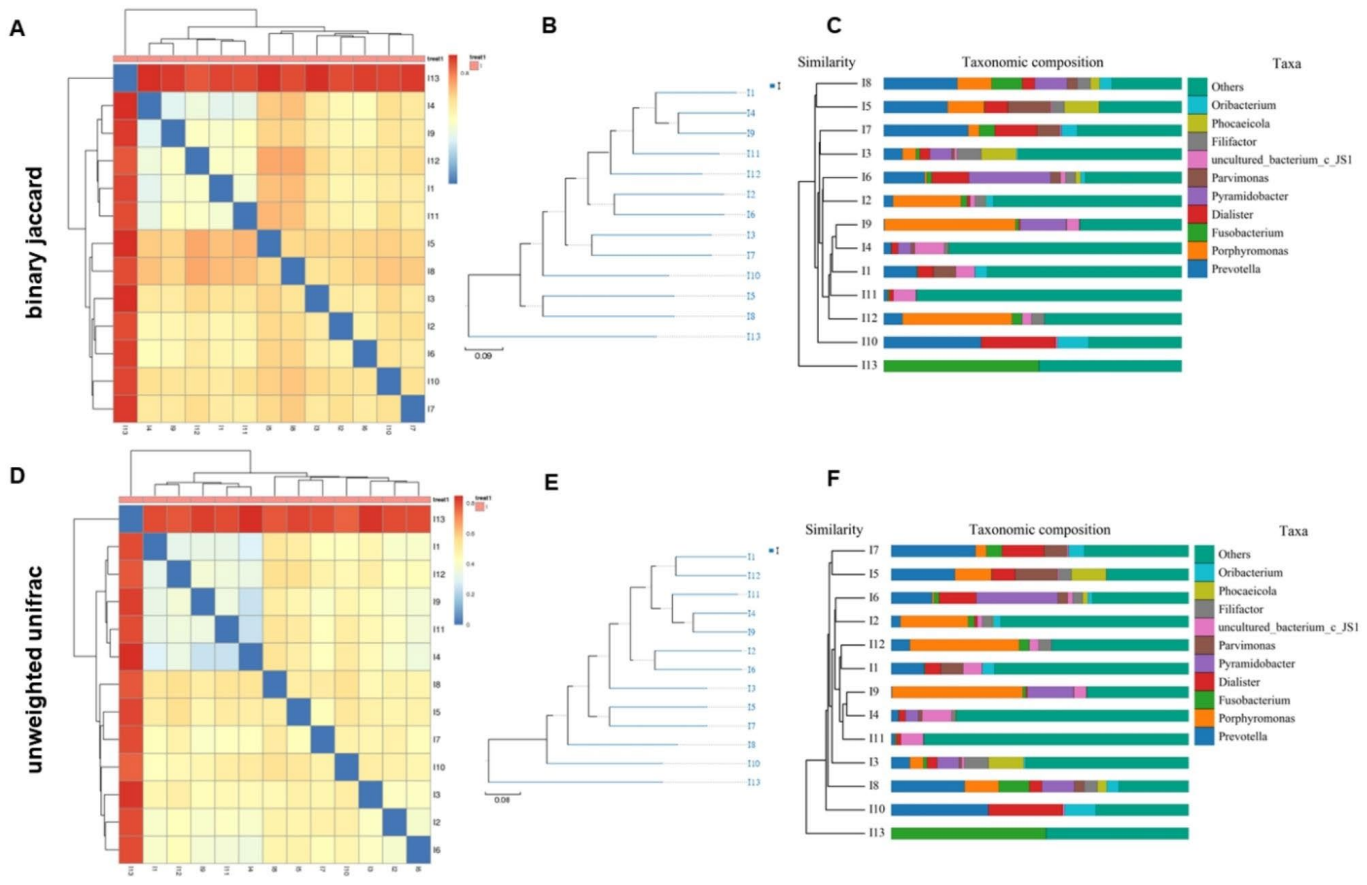
Inter-subject variability analysis. (**A**) Beta diversity analysis based on Binary-Jaccard. (**B**) UPGMA (Unweighted Pair-group Method with Arithmetic Mean) phylogenetic tree dendrogram based on Binary-Jaccard algorithm. (**C**) Abundance histogram based on Binary-Jaccard algorithm. (**D**) Beta diversity analysis based on the Unweighted Unifrac algorithm. (**E**) UPGMA phylogenetic tree dendrogram based on the Unweighted Unifrac algorithm. (**F**) Abundance histogram based on the Unweighted Unifrac algorithm. Note: The color in the lower left figure represents the color of the cluster tree sample group. The top right figure represents the top 10 species according to the table species abundance, others are classified as Others, and the species not noted are classified as Unclassified

Beta diversity analysis is used to compare the degree of difference among root canal samples. By the Binary-Jaccard algorithm, the difference between I13 and other samples is larger than those among other samples (Fig. 5A). To reveal the similarity and differences among the 13 primary endodontic samples, phylogenetic analysis using UPGMA (Unweighted Pair-group Method with Arithmetic Mean) and abundance histogram was performed (Fig. 5B C). The results showed that the 13 primary endodontic samples were divided into two groups, group A consisting of I1-I12 samples and group B containing I13. We used the Unweighted Unifrac algorithm to verify the results, and the consistent results were obtained (Fig. 5D-F). These data suggested that there was a wide inter-subject variability in the bacterial community in the root canals.

### Bacterial community in the primary root canal samples

**Fig. 6:**
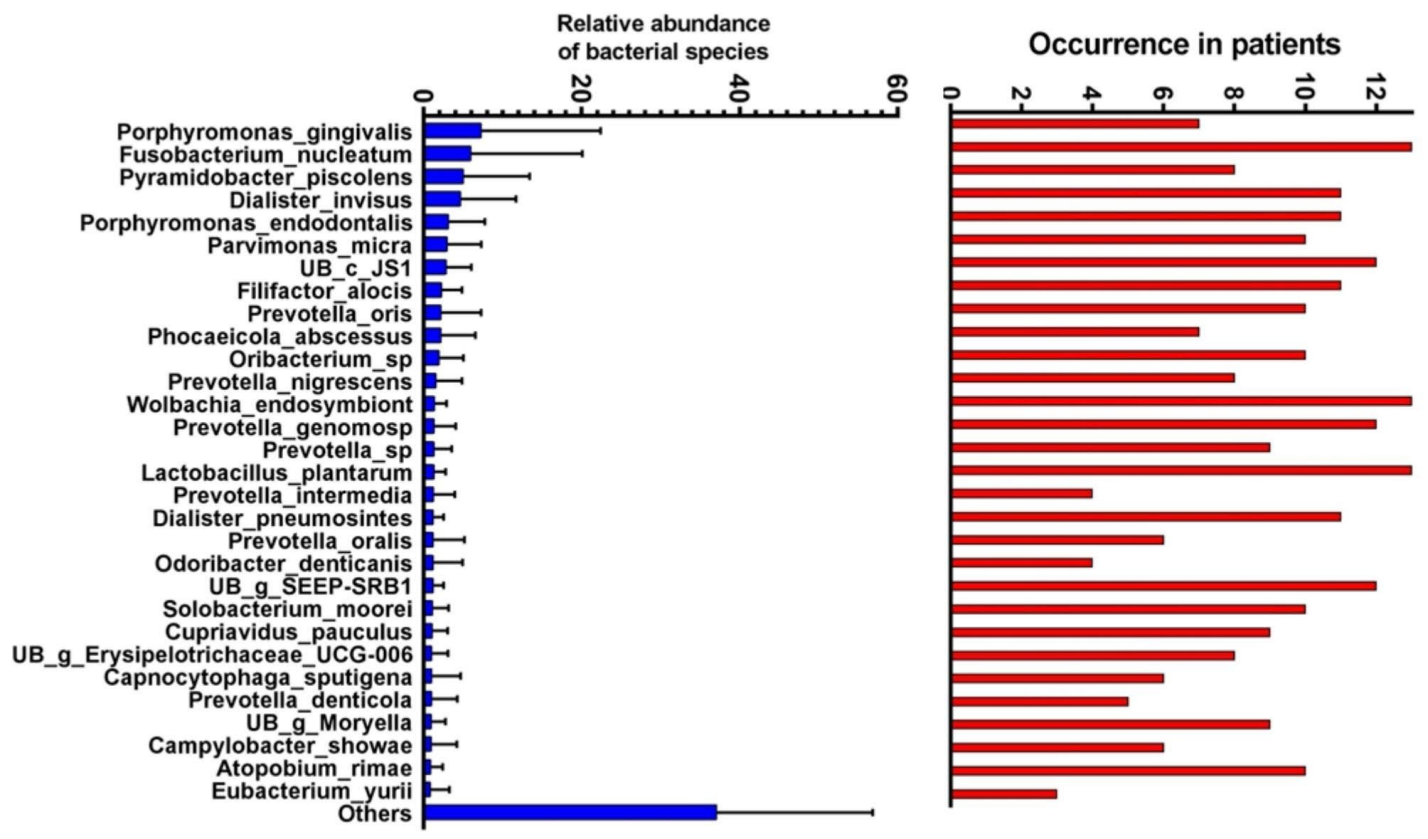
The occurrence frequency and abundance of bacterial species in the primary endodontic root canal samples

In the all 13 samples, the most abundant species was *Porphyromonas gingivalis*, followed by *Fusobacterium nucleatum*, *Pyramidobacter piscolens*, *Dialister invisus*, *Porphyromonas endodontalis*, *Parvimonas micra*, *UB c JS1*, *Filifactor alocis*, *Prevotella oris*, and *Phocaeicola abscessus*. *Fusobacterium nucleatum*, *Wolbachia endosymbiont*, *Lactobacillus plantarum*, *Klebsiella oxytoca*, and *Ralstonia pic*z*kettii*, were detected in all 13 patients (Fig. 6).

**Fig. 7:**
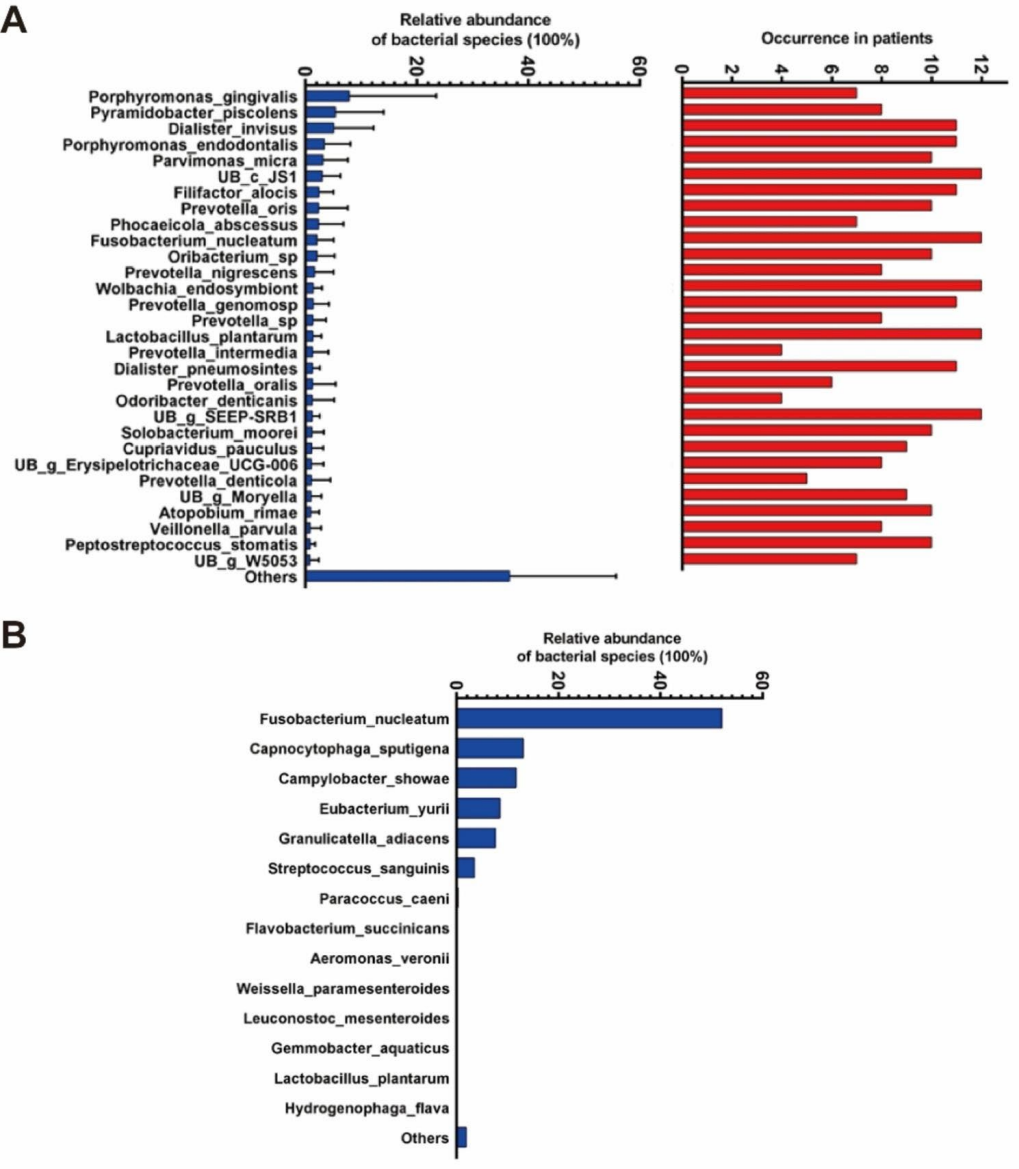
The occurrence frequency and abundance of bacterial species in the group A and group B of the primary endodontic root canal samples. (**A**) Abundance and detection rate of bacterial species in the Group A. (**B**) Abundance of species in the group B

In the group A, the most abundant species was *Porphyromonas gingivalis*, followed by *Pyramidobacter piscolens*, *Dialister invisus*, *Porphyromonas endodontalis*, *Parvimonas micra*, *UB c JS1*, *Filifactor alocis*, *Prevotella oris*, *Phocaeicola abscessus*, *Fusobacterium nucleatum*, *Oribacterium sp*, *Prevotella nigrescens*, and *Wolbachia endosymbiont*. A total of 35 species in common were detected in all the 12 patients in the group A, among them, *UB c JS1*, *Fusobacterium nucleatum*, *Wolbachia endosymbiont*, *Lactobacillus plantarum*, and *UB g SEEP-SRB1* were more abundant (Fig. 7A).

In the group B, the most abundant species was *Fusobacterium nucleatum*, followed by *Capnocytophaga sputigena*, *Campylobacter showae*, *Eubacterium yurii*, *Granulicatella adiacens*, *Streptococcus sanguinis*, and other species (Fig. 7B).

### Gender, age, duration of toothache-specific bacterial community associated with the patient group

**Fig. 8:**
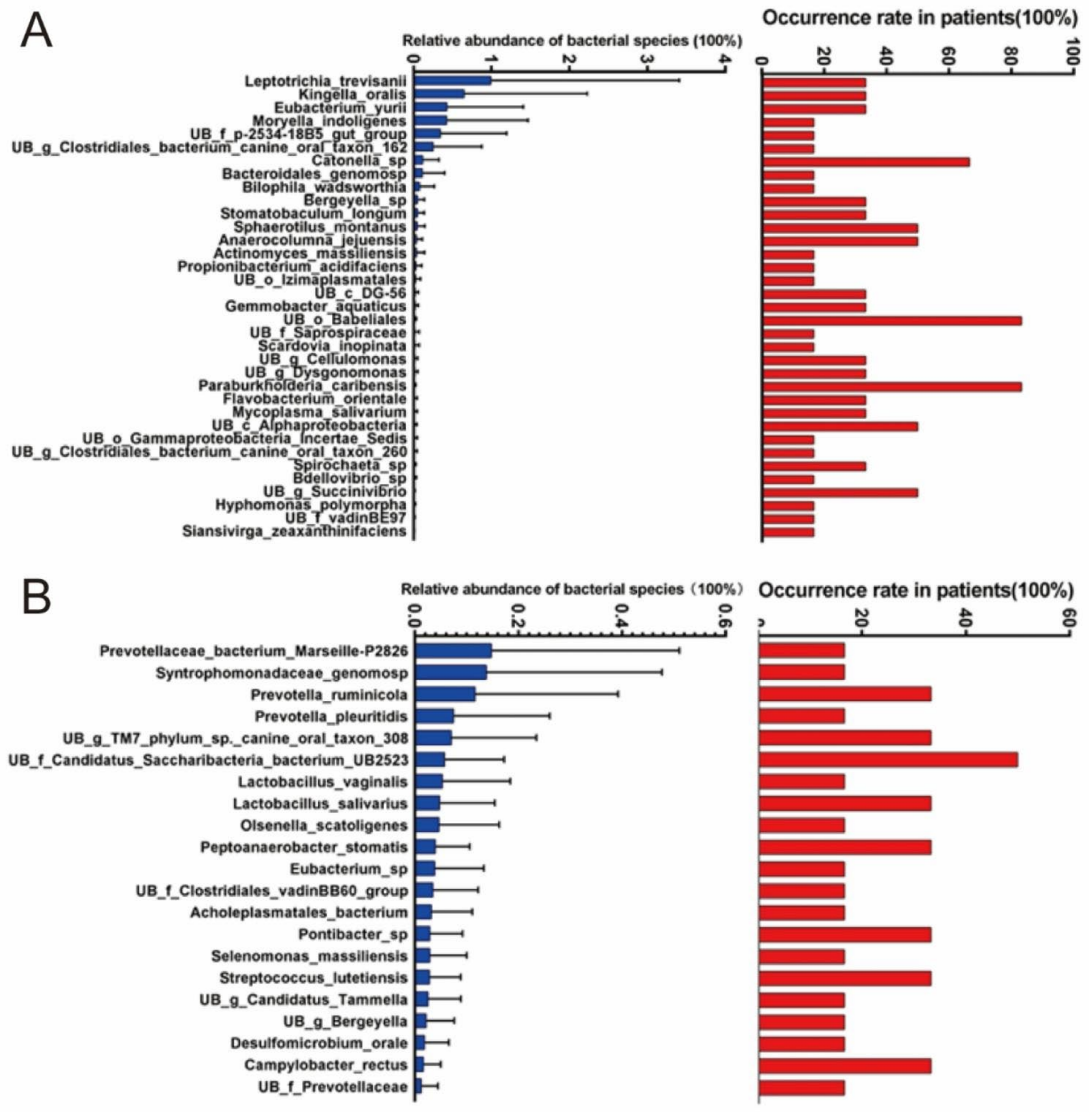
Gender-specific bacterial community associated with the patient group A. (**A**) Male patients. (**B**) Female patients

Because there were 12 samples in the group A and there was only one sample in the group B, we analyzed gender, age, duration of toothache-specific bacterial community associated with the patient group A. Analysis of gender-specific bacterial community associated with the patient group A showed that the male patient group had significantly higher abundance in *Paraburkholderia caribensis*, and *UB o Babeliales* (*P* = 0.002, 0.004) than those in the female group. *Leptotrichia trevisanii*, *Kingella oralis*, *Eubacterium yurii*, and *Moryella indoligenes* were only found in male patients (Fig. 8A). *Prevotellaceae bacterium Marseille-P2826*, *Syntrophomonadaceae genomosp*, *Prevotella ruminicola*, *Prevotella pleuritidis*, *UB g TM7 phylum sp. canine oral taxon 308*, *UB f Candidatus Saccharibacteria bacterium UB2523*, *Lactobacillus vaginalis*, and *Lactobacillus salivarius* were only found in the female patients (Fig. 8B).

**Fig. 9:**
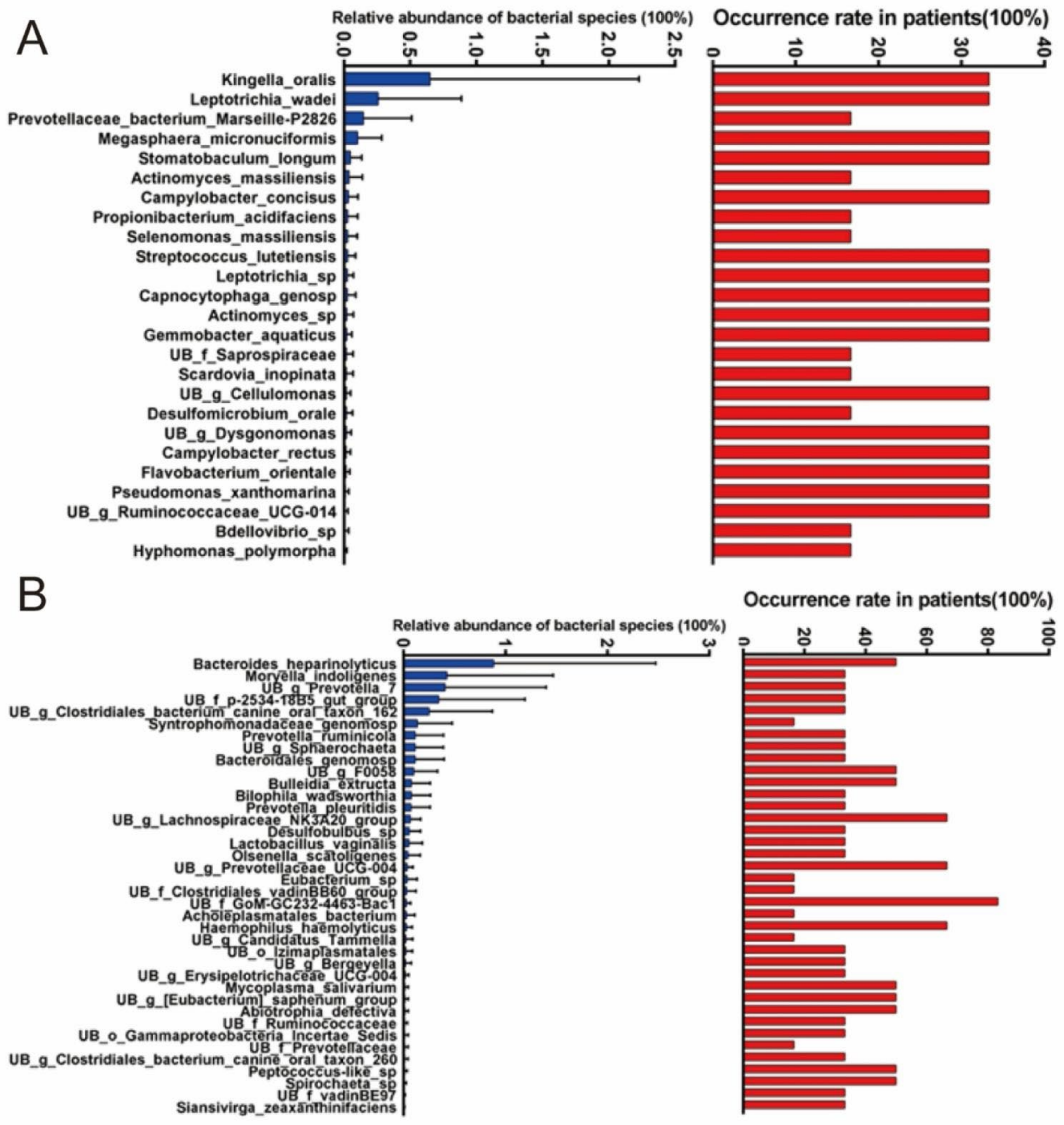
Age-specific bacterial community associated with the patient group A. (**A**) Younger patients. (**B**) Older patients

Analysis of age-specific bacterial community associated with the patient group A showed that there was no significant difference in the abundance of any species between the age-specific groups. *Kingella oralis*, *Leptotrichia wadei*, *Prevotellaceae bacterium Marseille-P2826*, *Megasphaera micronuciformis*, *Stomatobaculum longum*, *Actinomyces massiliensis*, *Campylobacter concisus*, *Propionibacterium acidifaciens*, *Selenomonas massiliensis*, and *Streptococcus lutetiensis* were only found in the young patient group (Fig. 9A). *Bacteroides heparinolyticus*, *Moryella indoligenes*, *UB g Prevotella 7*, *UB f p-2534-18B5 gut group*, *UB g Clostridiales bacterium canine oral taxon 162*, *Syntrophomonadaceae genomosp*, *Prevotella ruminicola*, *UB g Sphaerochaeta*, and *Bacteroidales genomosp* were only found in the old patient group (Fig. 9B).

**Fig. 10:**
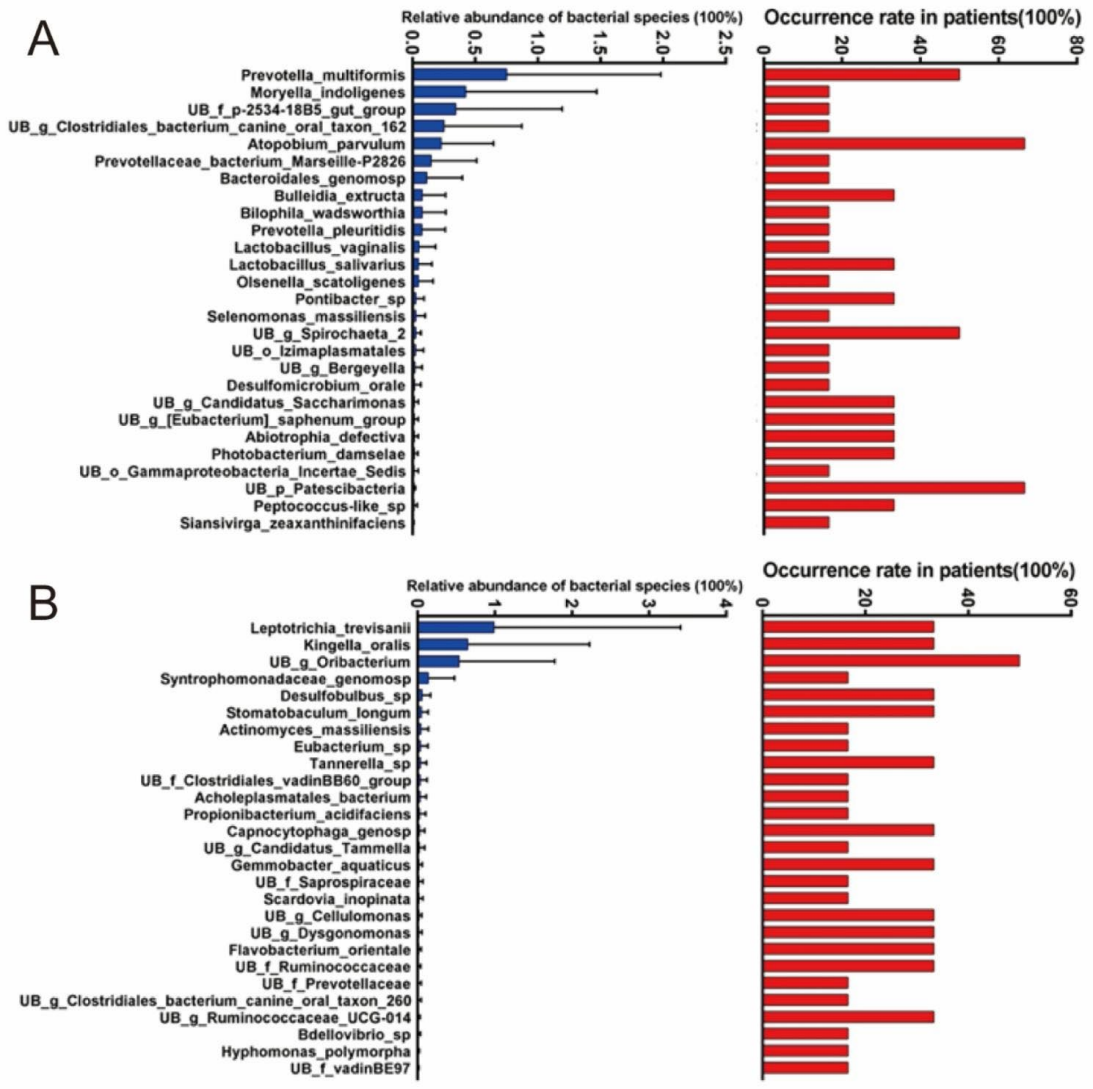
Duration-specific bacterial community associated with the patient group A. (**A**) Short duration of toothache. (**B**) Long duration of toothache

Analysis of duration-specific bacterial community associated with the patient group A showed that the patients with short duration of toothache had higher abundance in species *UB p Patescibacteria*, *UB c Gammaproteobacteria*, *UB g Pragia*, *UB p Omnitrophicaeota*, *UB f Burkholderiaceae*, and *Bacteroides vulgatus* than those with long duration of toothache (*P* = 0.01–0.05). *Prevotella multiformis*, *Moryella indoligenes*, *UB f p-2534-18B5 gut group*, *UB g Clostridiales bacterium canine oral taxon 162*, *Atopobium parvulum*, *Prevotellaceae bacterium Marseille-P2826*, *Bacteroidales genomosp*, and *Bulleidia extructa* were only found in the patient group with short-duration toothache (Fig. 10A). *Leptotrichia trevisanii*, *Kingella oralis*, *UB g Oribacterium*, *Syntrophomonadaceae genomosp*, *Desulfobulbus sp*, *Stomatobaculum longum*, *Actinomyces massiliensis*, *Eubacterium sp*, and *Tannerella sp* were only found in the patient group with long-duration toothache (Fig. 10B).

## Discussion

In the current study, we applied 16 S rDNA sequencing technology to analyze the microbial characteristics in the primarily infected root canal. We identified a total of 498 types of OTUs from 13 root canal samples with an average of 228, range from 56 to 343. These OTUs include a total of 40 Phyla, 63 Classes, 124 Orders, 180 Families, 265 Genera, and 359 Species. Significantly more OTUs are identified based on the 16 S rDNA direct-sequencing of the root canal samples than those identified by culture-based method because some microorganisms cannot grow under conventional conditions and are difficult to identify [26]. We found that there was inter-subject variability among 13 samples, and there was a large difference between I13 and I1-I12 through Beta diversity analysis. Based on the results, the 13 primary endodontic samples were divided into two groups, group A consisting of I1-I12 samples and group B containing I13. A total of 35 species in common were detected in all the 12 samples in the group A and only five OTUs were common in the all 13 root canal samples. This grouping excludes samples with a large degree of variation and reduces the error of subsequent analysis of group A, which has not been seen in previous studies. Therefore, there are a wide diversity and inter-subject variability in the bacterial community in the primary root canals. These results are consistent with previous studies [27, 28]. The most abundant phylum was Bacteroidetes, followed by Firmicutes, Proteobacteria, Fusobacteriota, Synergistota, Atribacteria, and Actinobacteria. The most abundant Class was Bacteroidia, followed by Clostridia, Gammaproteobacteria, Negativicutes, Fusobacteriia, Bacilli, Synergistia, and Tissierellia. The most abundant Order was Bacteroidales, followed by Veillonellales-Selenomonadales, Fusobacteriales, Synergistales, Lactobacillales, Peptostreptococcales-Tissierellales, and Clostridiales. The most abundant Family was Prevotellaceae, followed by Porphyromonadaceae, Veillonellaceae, Fusobacteriaceae, Synergistaceae, Lachnospiraceae, and Peptostreptococcaceae. The bacterial community structure maps at different taxonomic levels support that species composition varied from sample to sample, despite consistent disease diagnoses.

In the current study, *Fusobacterium nucleatum* was found in all root canal samples. The abundance of *Fusobacterium nucleatum* was high in the group A root canal samples and exceeded 50% in the group B sample. Our data indicated that it was the most variable and nearly the most abundant species in the bacterial community of the root canal samples. *Fusobacterium nucleatum* is a gram-negative obligate anaerobe and has a high detection rate in many oral infectious diseases, including root canal infections [29]. *Fusobacterium nucleatum* can bind closely with early colonizing bacteria, and then copolymerize various pathogenic bacteria with the help of various resins secreted by itself, thus enhancing pathogenicity. Therefore, *Fusobacterium nucleatum* is considered the “bridge bacterium” in the process of disease development [30, 31]. *Fusobacterium nucleatum* plays an important role in the biofilm formed by initial infection around the root tip [17, 32]. In addition, *Fusobacterium nucleatum* destroys bone tissue by secreting virulence factors and releasing endotoxins and metabolites. *Fusobacterium nucleatum* is also closely related to the occurrence and development of respiratory diseases, colorectal cancer, and premature delivery in pregnant women [33, 34]. It is likely that *Fusobacterium nucleatum* is a major microbial factor responsible for root canal infection and systematic diseases through multiple mechanisms.

It is consistent with previous study that both *Prevotella* and *Porphyromonas gingivalis* are isolated from infected root canals [35]. In the current study, although the most abundant Genus was *Prevotella*, followed by *Porphyromonas* and *Fusobacterium*, *Porphyromonas gingivalis* is the most abundant and common species in the group A of 12 root canal samples. However, this is different from previous study that shows *Enterococcus faecalis* is more prevalent than *Porphyromonas gingivalis* in the primary endodontic infection [36]. This is also different from the findings based on Chinese patients living in Beijing, where primary endodontic infection is mainly associated with *Burkholderia cepacia*, *Actinomyces*, *Aranicola spp*. and *Streptococcus sanguinis* [37], and Chinese patients in Shanghai, where *Parvimonas micra*, *Porphyromonas endodontalis*, *Tannerella forsythia*, *Prevotella intermedia* and *Porphyromonas gingivalis* are the most prevalent taxa and were often concomitant [38]. It seems that the predominant species responsible for root canal infections are associated with geographical or ethnic groups.

Specific strains may be associated with different clinical and radiological characteristics of patients. It has been found that *Porphyromonas gingivalis* is associated with previous pain and pain caused by taping, and the presence of *Enterococcus faecalis* and *Fusobacterium nucleatum* is associated with periapical lesions > 3 mm [9]. Both *Fusobacterium nucleatum* and *Porphyromonas gingivalis* may contribute to the immunopathogenesis of apical periodontitis although their LPS presented different patterns of activation against macrophages as seen by the IL-1beta and TNF-alpha production [33]. Therefore, *Porphyromonas gingivalis* is likely a major microbial factor responsible for root canal infection in a Chinese subpopulation in Shiyan, Hubei Province.

In the study, we have analyzed gender, age, duration of toothache-specific bacterial community associated with the patient group to identify bacterial factors contributing to clinical symptomatology of root canals. For instance, long root canal infection decreases the ability of the body to regenerate the dentin-pulp complex [39]. Our data showed that the male patient group had significantly higher abundance in three species, *Paraburkholderia caribensis*, *UB o Babeliales*, *UB g Succinivibrio* and significantly lower abundance in *Parvimonas micra* than those in the female group. The patients with short duration of toothache had higher abundance in species *UB p Patescibacteria*, *UB c Gammaproteobacteria*, *UB g Pragia*, *UB p Omnitrophicaeota*, *UB f Burkholderiaceae*, and *Bacteroides vulgatus* than those with long duration of toothache. We do not find significant difference in the abundance of any species between the age-specific groups. The abundance of these identified species are very low, compared with those abundant species, *Porphyromonas gingivalis*, *Pyramidobacter piscolens*, *Dialister invisus*, *Porphyromonas endodontalis*, *Parvimonas micra*, *UB c JS1*, *Filifactor alocis*, *Prevotella oris*, *Phocaeicola abscessus*, *Fusobacterium nucleatum*, *Oribacterium sp*, *Prevotella nigrescens*, and *Wolbachia endosymbiont*, and also compared with those common species, *UB c JS1*, *Fusobacterium nucleatum*, *Wolbachia endosymbiont*, *Lactobacillus plantarum*. We speculate that these gender, age, duration of toothache-specific bacterial community associated with the patient group are less significant than those abundant and common species. Their roles in the clinical symptomatology of root canals remain to be investigated.

However, there are some limitations to our study. First, due to constraints, we only collected samples from primary periapical periodontitis. If samples from other diagnosis such as secondary periapical periodontitis could be collected, it would be better to reflect the correlation between bacteria and the disease. Second, Samples were collected using the method of three consecutive collections with sterile paper point, a widely used collection technique in root canals. However, due to the small surface area of paper point, only limited samples can be collected and the bacterial abundant in the root canal cannot be accurately reflected. In addition, the root canal system is so complex that paper point cannot reach the sites where bacteria are concealed. Therefore, it is necessary to explore a more accurate method for bacterial sampling in root canals. Third, more clinical samples and experiments are needed to verify the correlation with bacterial diversity and clinical symptoms. In this way, we could gain more accurately understanding to the role of bacteria in the development of diseases, and can also provide better help to clinicians.

## Conclusion

In conclusion, there are a wide diversity and inter-subject variability in the bacterial community in the primary root canals. The bacterial community at different taxonomic levels varies from sample to sample, despite consistent disease diagnoses. While *Porphyromonas gingivalis* is the most abundant species, *Fusobacterium nucleatum* was the most variable species in the bacterial community of the root canals in the patients subjected to root canal treatment in Shiyan, Hubei Province. There is gender, duration-specific differences in the bacterial species.

### Electronic supplementary material

Below is the link to the electronic supplementary material.

Supplementary Material 1

## Data Availability

The rawdata in the study had been submitted to GenBank, the relevant accession numbers is: file SUB13453232: OR045419 - OR045824.
