# Supplementary figures and images for: Bacterial diversity in primary infected root canals of a Chinese cohort: analysis of 16 S rDNA sequencing
Source: BMC Oral Health. 2023 Nov 27;23:932. doi: 10.1186/s12903-023-03618-3 (PMC10680180; doi:10.1186/s12903-023-03618-3)

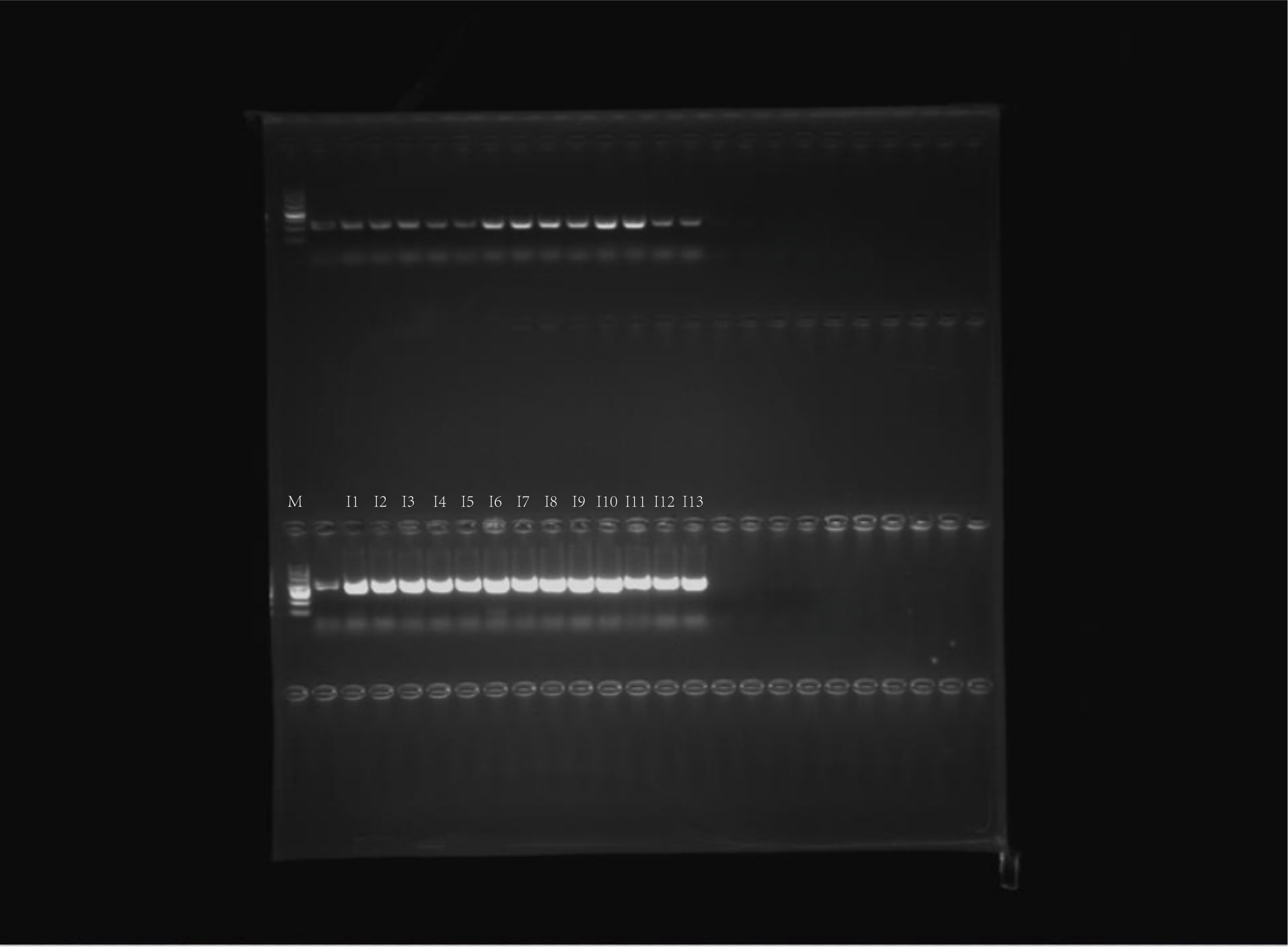

Supplement: Supplementary file 1 — Supplementary Material 1 [file 12903_2023_3618_MOESM1_ESM.png]
